# Development of a Spinal Cord Injury Model Permissive to Study the Cardiovascular Effects of Rehabilitation Approaches Designed to Induce Neuroplasticity

**DOI:** 10.3390/biology10101006

**Published:** 2021-10-07

**Authors:** Liisa Wainman, Erin L. Erskine, Mehdi Ahmadian, Thomas Matthew Hanna, Christopher R. West

**Affiliations:** 1 Centre for Chronic Disease Prevention and Management, Faculty of Medicine, University of British Columbia, Kelowna, BC V1V 1V7, Canada; lwain27@student.ubc.ca (L.W.); erin.erskine@ubc.ca (E.L.E.); mahmadia@student.ubc.ca (M.A.); tmhanna7@student.ubc.ca (T.M.H.); 2 International Collaboration of Repair Discoveries (ICORD), University of British Columbia, Vancouver, BC V5Z 1M9, Canada; 3 School of Kinesiology, Faculty of Education, University of British Columbia, Vancouver, BC V6T 1Z1, Canada; 4 Department of Biology, Faculty of Science, University of British Columbia Okanagan, Kelowna, BC V1V 1V7, Canada; 5 Department of Cell and Physiological Sciences, Faculty of Medicine, University of British Columbia, Vancouver, BC V6T 1Z3, Canada

**Keywords:** cardiovascular, contusion, neuroplasticity

## Abstract

**Simple Summary:**

People living with high-level spinal cord injury experience worse cardiovascular health than the general population. In most spinal cord injuries, there are some remaining functioning pathways leading from the brain through the spinal cord to the organs and muscles, but not enough to sustain normal levels of function. Recently, therapies that aim to increase the strength of connections in these remaining pathways have shown great potential in restoring walking, hand, and breathing function in the spinal cord injured population. In order to test these therapies for their effects on cardiovascular function, we developed a new type of spinal cord injury rat model that spares enough pathways for these therapies to act upon but still produces measurable reductions in heart and blood vessel function that can be targeted with interventions/treatments.

**Abstract:**

As primary medical care for spinal cord injury (SCI) has improved over the last decades there are more individuals living with neurologically incomplete (vs. complete) cervical injuries. For these individuals, a number of promising therapies are being actively researched in pre-clinical settings that seek to strengthen the remaining spinal pathways with a view to improve motor function. To date, few, if any, of these interventions have been tested for their effectiveness to improve autonomic and cardiovascular (CV) function. As a first step to testing such therapies, we aimed to develop a model that has sufficient sparing of descending sympathetic pathways for these interventions to target yet induces robust CV impairment. Twenty-six Wistar rats were assigned to SCI (n = 13) or naïve (n = 13) groups. Animals were injured at the T3 spinal segment with 300 kdyn of force. Fourteen days post-SCI, left ventricular (LV) and arterial catheterization was performed to assess in vivo cardiac and hemodynamic function. Spinal cord lesion characteristics along with sparing in catecholaminergic and serotonergic projections were determined via immunohistochemistry. SCI produced a decrease in mean arterial pressure of 17 ± 3 mmHg (*p* < 0.001) and left ventricular contractility (end-systolic elastance) of 0.7 ± 0.1 mmHg/µL (*p* < 0.001). Our novel SCI model produced significant decreases in cardiac and hemodynamic function while preserving 33 ± 9% of white matter at the injury epicenter, which we believe makes it a useful pre-clinical model of SCI to study rehabilitation approaches designed to induce neuroplasticity.

## 1. Introduction

Spinal cord injury (SCI) is a debilitating condition which, in addition to inducing sensorimotor dysfunction, also impairs autonomic function. Cardiovascular disease (CVD) has emerged as the primary cause of morbidity and mortality for individuals living with SCI [1]. SCI-induced dysregulation of the cardiovascular (CV) system occurs primarily as a result of altered descending control of sympathetic preganglionic neurons (SPNs). In turn, such reduced medullary input to SPNs causes a host of CV complications including resting hypotension, orthostatic hypotension (OH; sudden decrease in BP upon changing posture), autonomic dysreflexia (AD; sudden episodic hypertension accompanied by reflex bradycardia), and left-ventricular systolic function, which precipitate the early development of CVD [2].

In addition to changes in CV control, the sympathetic nervous system undergoes remarkable plasticity. These changes include decreased synaptic density accompanied by an increase in the number of inhibitory synapses rostral to the injury [3]. Caudal to the lesion, increased synaptogenesis [4] and changes in SPN morphology occurs, including increased arborization of SPNs [5] and axonal sprouting [6]. Historically, such sympathetic neuroplasticity has largely been considered detrimental due to the association of such plasticity with the expression of autonomic dysreflexia, immune suppression and neuropathic pain following SCI [7,8,9].

In the wider field of SCI, a number of recent promising interventions have been proposed that seeks to either leverage plasticity for functional benefit or alter such plasticity to offset functional decline. For example, the delivery of acute intermittent hypoxia (AIH) has been shown to enhance synaptic input onto spinal motor neurons and increase spinal excitability, both of which increase synaptic strength [10,11] and subsequently improve motor output in the acute [12,13] and chronic settings post-SCI [14,15]. Activity based therapy (ABT) is another intervention that has been demonstrated to facilitate the recovery of specific tasks (i.e., swimming) [16,17], hind-limb [18,19], and forelimb function [20,21]. These functional benefits are associated with increased spinal brain-derived neurotrophic factor (BDNF) levels and synaptic plasticity [22]. In all the aforementioned studies, the benefits of these therapies have been demonstrated in incomplete models of cervical and/or lower-thoracic (i.e., T9/10) SCI, wherein the injury is either not severe enough to induce CV dysfunction (i.e., the incomplete cervical models) or below the spinal level at which innervation to the key vascular beds and heart occurs (i.e., the low thoracic T9/T10 models).

For the CV system, a number of rat models have been developed to study the CV consequences of SCI, as well as the efficacy of various therapeutics. Two of the models that have received most traction are the T3/T4 complete transection model or a very severe midline contusion injury [23,24], though others also exist [25]. Both transection and severe contusion injuries (i.e., 400 kdyn contusion model) have been effective in producing changes to the CV function that mimic those observed clinically with high-lesion SCI, such as the presence of pronounced hypotension, reduced systolic cardiac function, and the presence of autonomic dysreflexia and orthostatic intolerance [23,26,27,28,29]. However, because these models either severed all pathways (in the case of transection injuries) or preserved such few medullary sympathetic pathways (i.e., <5% in the case of severe contusion) they are likely to be inappropriate to test the application of interventions designed to strengthen spinal sympathetic pathways. Indeed, in the few studies that have investigated the effect of ABT on CV function using such models post-SCI it has been shown that ABT was ineffective in restoring blood pressure control and systolic cardiac function, presumably because there were not sufficient bulbo-spinal sympathetic pathways left for ABT to target [27]. Instead, any benefits of ABT in these settings appear to be limited to the peripheral circulation and/or muscle.

Here, we present an in vivo and histological validation of a new moderately severe mid-line contusion injury model at the T3 level that we believe demonstrates an excellent balance between sparing sufficient bulbo-spinal sympathetic pathways that can be targeted with therapies, yet still induces a consistent and measurable decline in CV function that mimics that which occurs clinically. We propose that this model also more accurately reflects the changing demographic observed clinically, where the number of individuals with neurologically incomplete high-level injuries now outnumber those with neurologically complete injuries.

## 2. Materials and Methods

### 2.1. Ethical Approval

All procedures were conducted in accordance with the Canadian Council for Animal Care. Ethical approval was also obtained from the University of British Columbia (ACC-A18-0344).

### 2.2. Experimental Design

A total of 26 male Wistar rats (Charles River Laboratories, 11 ± 1 week old) were assigned to either SCI (n = 13) or naive (n = 13) groups. Study endpoint was conducted at 2 weeks post-SCI. This timeframe was selected as reductions in BP fully manifest by day 6 [29] and cardiac dysfunction is present immediately following injury [30,31] and persists into the chronic phase (i.e., 12 weeks post-SCI) [31,32]. Following in vivo measures, 5 SCI animals were randomly selected for standard spinal cord immunohistochemistry quantification of the injury site, and the 3 animals had their spinal cords harvested and cut in the longitudinal axis to visualize descending catecholaminergic and serotonergic bulbo-spinal projections, both of which are known to play a key role in CV control in the chronic phase post-SCI [24,33].

### 2.3. Spinal Cord Injury Surgery

**Figure 1:**
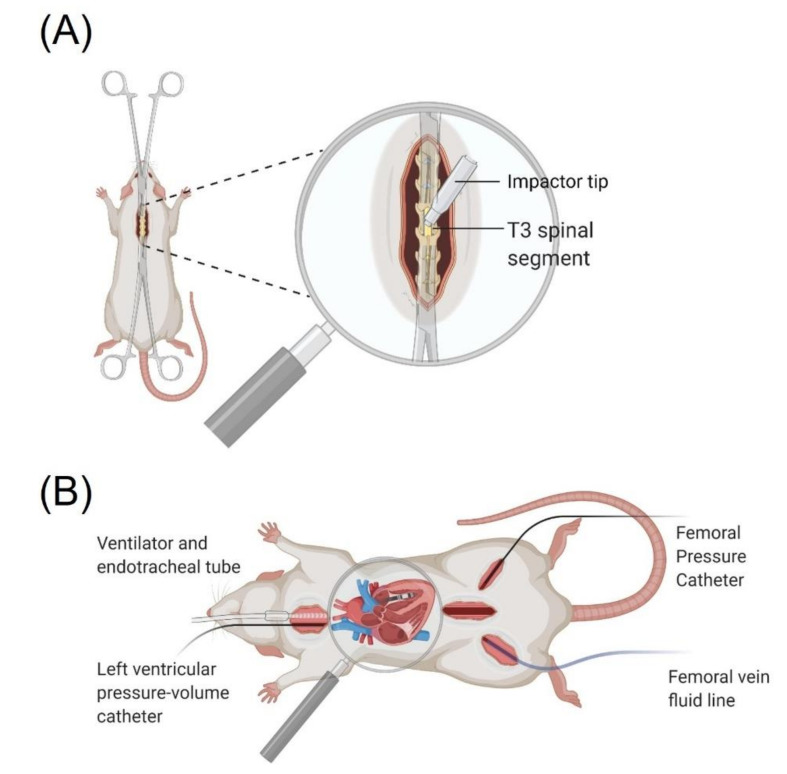
(**A**) Rodent SCI surgery setup depicting laminectomy and contusion injury method. (**B**) In vivo terminal preparation consisting of an endotracheal tube and ventilator, left ventricular pressure-volume catheter, femoral artery pressure catheter, and femoral venous line.

Rats were prepared for spinal cord contusion surgery as described in previous studies [24,26,34,35]. The surgical preparation is depicted in Figure 1A. Briefly, on the day of SCI animals were anesthetized (5% isofluorane chamber induction, maintenance on 1.5–2% isofluorane; Piramal Critical Care, Bethlehem, PA, USA) and administered enrofloxacin (10 mg/kg; Bayer Animal Health, Shawnee, KS, USA), buprenorphine (0.5 mg/kg; Ceva Animal Health, Cambridge, ON, Canada) and warmed lactated ringer’s solution (5 mL subcutaneously; Baxter Corporation, Portland, OR, USA). A dorsal midline incision was made and paraspinal musculature was bluntly dissected to expose C8–T5 spinous processes. A T3 laminectomy was performed exposing the T3 dura. Rodents were then transported and mounted on a plastic staging platform where the T2 and T4 spinous processes were stabilized with curved tip clamps. Using a high-definition camera secured to the mounting frame, the custom impactor tip (3 mm; Infinite Horizons (IH) Impactor; Precision Systems and Instrumentation, Fairfax Station, VA, USA) was adjusted to track midline over the T3 dura. The impactor tip was dropped on the cord with 300 kdyn of predefined force (316 ± 14 kdyn force, 1673 ± 128 mm displacement, 124 ± 5 mm/s velocity). The muscle and the skin incisions were closed with 4-0 coated vicryl (Ethicon, Somerville, MA, USA). Velocity, force of impact, and distance travelled by the impactor were recorded. Animals were recovered in an incubator for 30 min at 37 °C 50% humidity and received a subsequent 5 mL lactated ringer’s solution before they were returned to their home cages.

### 2.4. Post-Surgical Care

For 4 days post injury, animals were administered subcutaneous lactated ringers (3× per day, 5 mL), buprenorphine (3× per day, 0.02 mg/kg) and enrofloxacin (1× per day, 10 mg/kg). Bladders were manually voided 4× per day until spontaneous voiding was regained (4–6 days post-injury). Animals were pair-housed on oat bedding with rubber matting to prevent the ingestion of woodchips due to opioid-induced pica and to aid in mobility. Animals were provided a supportive diet consisting of Hydrogel (ClearH_2_O), fruit, spinach, and cereal until mobility was regained and pica subsided.

### 2.5. Outcome Surgery

At 14 days post-SCI, echocardiography was performed to assess left ventricular (LV) structure, cardiac catheterization was performed to model LV pressure-volume relationships and assess LV contractility, arterial catheterization was performed to assess blood pressure and a venous line was placed for intravenous fluid administration to maintain acid-base balance (Figure 1B).

For the terminal in vivo assessments, animals were anesthetized with intraperitoneal urethane (1.6 ± 0.4 mg/kg; Sigma-Aldrich, St. Louis, MO, USA). Animals were instrumented with a rectal thermometer and all procedures were performed on a heating pad (RightTemp; Kent Scientific, Torrington, CT, USA) to maintain core body temperature at 37 ± 0.5 °C. Transthoracic echocardiography was used to obtain B-mode parasternal long axis images to measure LV volumes (Vevo 3100; VisualSonics, Toronto, ON, Canada). Next, the rat was placed supine, and a midline incision was performed from mandible to manubrium. Sternohyoid muscle was bluntly dissected then trachea and right common carotid artery (CCA) isolated. A tracheostomy was performed, an endotracheal tube was secured, and the animal was ventilated on 100% O_2_ (VentElite; Harvard Apparatus, Holliston, MA, USA) using a standard tidal volume and breathing frequency calculation based off the animal`s mass [36]. The CCA was pierced, and a 1.9-French pressure-volume (PV) admittance catheter (Transonic Scisense, Ithaca, NY, US) advanced into the LV [36]. Bilateral incisions along the inguinal ligament were performed and the femoral artery and vein were isolated. A 1.6-French pressure catheter (Transonic Scisense, Ithaca, NY, USA) was placed into the left femoral artery and advanced into the abdominal aorta for collection of hemodynamic data. The right femoral vein was cannulated with a fluid delivery line (PE50 tubing) for constant infusion of lactated ringer’s solution throughout the experiment (1.7 mL/kg/h; Pump 11 Elite, Harvard Apparatus, Holliston, MA, USA). Finally, a ventral laparotomy was performed and inferior vena cava isolated to perform inferior vena cava occlusions (IVCOs) which enables venous return to be reduced and the slope of the end-systolic pressure-volume relationship to be obtained. The slope of this relationship is end-systolic elastance and is the reference standard for load-independent LV contractility [36].

Following the completion of instrumentation, the animal was allowed to stabilize for 15 min prior to the collection of a 5 min baseline for the assessment of hemodynamics and cardiac function. An IVCO was then performed to assess end-systolic elastance.

### 2.6. Ethanasia and Tissue Processing

Following the completion of all in vivo measures, 8 animals were selected at random for immunohistological preparation. Rats were perfused transcardially with 200–300 mL of 0.1 M phosphate-buffered saline (PBS; Sigma-Aldrich, St. Louis, MA, USA) and fixed with 400–500 mL 4% paraformaldehyde (PF; Sigma-Aldrich, St. Louis, MA, USA). Lesion sites (±4 mm from epicenter; T1–T5 segments) were dissected following perfusion and stored in PF for no more than 48 h followed by at least 24 h in 10% sucrose before being flash frozen in Shandon Cryomatrix (Thermo Scientific, Cat: 67-690-06, Waltham, MA, USA) and stored at −80 °C.

### 2.7. Data Analysis

Echocardiography indices were obtained from an average of 3 end-systolic and end-diastolic images from each animal and used to correct PV estimates of volumes.

All PV indices were analyzed using the PV loop analysis software in Labchart8 (AD Instruments). The following measures of LV systolic function were averaged across the final 60 s of baseline data: stroke volume (SV; calculated as end diastolic volume [EDV]-end systolic volume [ESV]), ejection fraction (EF; calculated as SV/EDV×100%), end-systolic pressure (Pes), the maximal rate of rise of the LV pressure (dP/dt_max_), dP/dt_max_ normalized to end-diastolic volume (dP/dt_max_−EDV), stroke work (SW; area inside the PV loop), stroke work index (SWI; SWI = SW/g), cardiac output (CO = SV·HR), cardiac index (CI; CI = CO/g), The following indexes of diastolic function were also measured from the same loops: end-diastolic pressure (Ped), maximal rate of fall of the LV pressure waveform (dP/dt_min_), and the time constant of LV pressure decay during isovolumetric relaxation. Hemodynamic indices systolic blood pressure (SBP), diastolic blood pressure (DBP), pulse pressure (PP) (calculated as PP = SBP–DBP), heart rate (HR), mean arterial pressure (MAP; calculated as MAP = 1/3SBP + 2/3 DBP) and systemic vascular resistance (SVR; SVR = MAP/CO) were extracted from the same 60 s.

Load-independent indices of LV contractility were calculated from one IVC occlusion. One 10-s section of the IVC occlusion was selected and loops that occurred during an expiration were removed to prevent respiratory-induced changes in intrathoracic pressure right-shifting the PV loop. Preload-recruitable stroke work (PRSW) was evaluated as the slope of the linear regression of SW and EDV. End-systolic elastance (Ees) was taken as the slope of the end-systolic pressure-volume relationship. dP/dt_max_-EDV was calculated as the slope of the linear regression of dP/dt_max_ to EDV.

Arterial elastance (Ea) was calculated as Ea = Pes/SV. Ea/Ees was calculated as the quotient of Ea divided by Ees.

### 2.8. Immunohistochemistry

Spinal cords were cut using a cryostat (Leica, CM3050s, Wetzlar, Germany) in either the transverse (n = 5) or longitudinal (n = 3) plane. Transverse sections were cut at 10 µm thickness with an inter-section distance of 1 mm. Longitudinal sections (n = 3) were cut at 10 µm thickness and with an inter-section distance of 600 µm.

Slides were thawed and dried at room temperature for 20 min then a hydrophobic barrier was drawn. Slides were rehydrated with 3 10-min washes in PBS followed by incubation in blocking solution (10% normal donkey serum) in PBS-Tx-Azd for 45 min. Slide were then incubated with primary antibodies over night. The next day the tissue was washed three times (15 min each) with PBS, incubated with secondary antibodies for 2 h, and then washed with PBS three times (15 min each). Finally, the slides were cover-slipped using ProLong Gold antifade mounting medium (Invitrogen, LSP36930, Waltham, MA, USA).

For transverse sections primary antibodies were used as follows; mouse GFAP (Glial fibrillary acidic protein; 1:1000, Sigma; G3893, Waltham, MA, USA), chicken polyclonal MBP (Myelin basic protein; 1:1000, Aves Labs; MBP), guineapig NeuN (Neuronal nuclei; 1:500, Sigma; ABN90P, St. Louis, MA, USA). The following secondaries were used; donkey anti-mouse Cy3 (1:800, Jackson Immunoresearch; 705-166-147, West Grove, PA, USA), donkey anti-chicken pAb Alexa647 (1:800, Jackson Immunoresearch; 7056-606-148, West Grove, PA, USA), donkey anti-guineapig DyLight405 (1:800, Jackson Immunoresearch; 711-475-152, West Grove, PA, USA).

For longitudinal slides primary antibodies were used as follows; sheep TH (tyrosine hydroxylase; 1:200, EMD Milipore; AB1542, Burlington, VT, USA), rabbit 5-HT (5-hydroxytryptamine; 1:2000, Immunostar; 20080. Hudson, NY, US). The following secondaries were used; Donkey anti-sheep Cy3 (1:200, Jackson Immunoresearch; 713-166-147, West Grove, PA, USA) and donkey anti-rabbit DyLight488 (1:1000, Abcam; ab96899, Cambridge, UK).

Immunofluorescence imaging was performed using an Axio Imager M2 microscope (Zeiss, Oberkochen, Germany) with an Axiocam 705 mono camera (Zeiss, Oberkochen, Germany) using ZEN 2 Blue software (Zeiss, Oberkochen, Germany). Images were digitally processed using Zen 2 Blue software (Zeiss, Oberkochen, Germany).

Analysis was performed in ImageJ (ImageJ, Rockville, MD, USA). Lesion area and white matter sparing were quantified every 400 µm from 2.0 mm rostral to 2.0 mm caudal to the injury epicenter. The injury epicenter section was based on the section with the least intact GFAP signal. Lesion area was manually outlined based on the following definition: GFAP-negative or GFAP-positive area with disrupted or abnormal cytoarchitecture. Care was taken to avoid inclusion of any artifacts. Myelin preservation (i.e., white matter sparing) was estimated by manually outlining MBP-positive area with normal or near-normal cytoarchitecture. Lesion volume was then calculated according to the following formula: Volume = Σ (area · section thickness · number of sections between samples) [24].

For longitudinal sections, images were imported into ImageJ (ImageJ, Rockville, MD, USA) and converted to 8-bit. The backgrounds were then subtracted, and for each stain, the images were set to a threshold only including pixels with intensity values from 20–255. The analyzed regions were selected by tracing the epicenter and selecting 2 × 1 mm rectangles 0.5 mm rostral and caudal to the border of the lesion. After measuring the positive pixel density of the enclosed areas, the density of the caudal area was divided by the density of the rostral area to calculate the percent difference. The relative density of an anterior, posterior and central section of the cord for each animal was calculated and expressed as means and standard deviations calculated from 3 animals.

### 2.9. Statistics

Between-group differences in all in vivo physiological outcomes were analyzed using an independent samples *t*-test in SPSS (IBM SPSS Statistics, Chicago, IL, USA). Data are expressed as means ± standard deviation. Statistical significance was set at *p* < 0.05. Graphical representations of in vivo data were produced in MATLAB (MathWorks, Natick, MA, USA) and Prism (GraphPad Prism, San Diego, CA, USA). Histological images were produced in Zen Image Processing (Zeiss, Oberkochen, Germany).

## 3. Results

**Figure 2:**
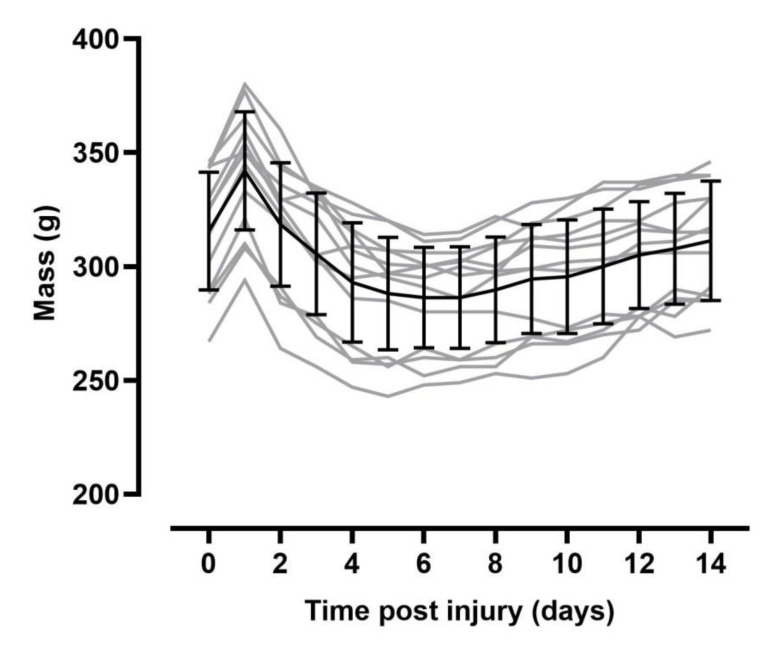
Body mass with time pre/post-injury. Note there were no significant differences in body mass across the 14-day study period. Black lines indicate mean mass ± SD, grey lines represent individual animals body mass.

At study termination SCI rats were significantly lighter than naïve animals (*p* = 0.014; Figure 2) but there was no significant difference in body mass of SCI animals at day 14 post-injury vs. pre-injury (*p* = 0.154).

### 3.1. Resting Hemodynamics Are Impaired in T3 300 kdyn SCI Rats

**Figure 3:**
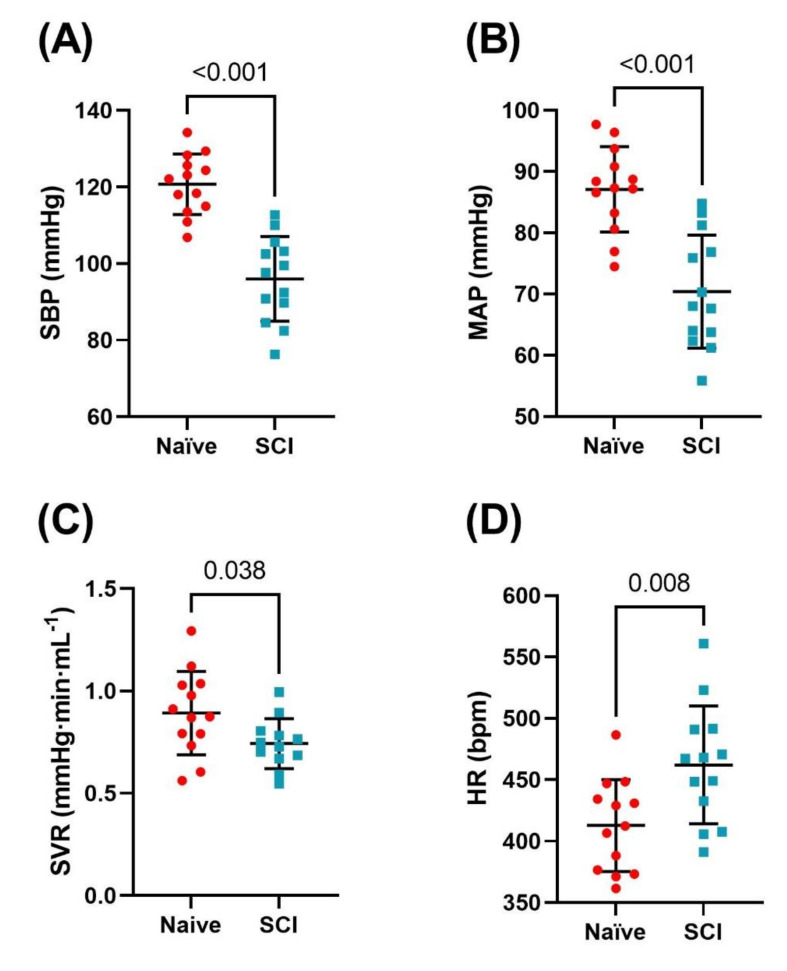
Comparison of resting hemodynamic indices of naïve (n = 13) and spinal cord injured (SCI) rats (n = 13) two weeks post-injury. Bars represent the means and standard deviations overlaid with individual data. (**A**) Systolic blood pressure (SBP) was significantly lower in SCI compared to naïve animals (25 ± 4 mmHg, *p* < 0.001). (**B**) Mean arterial pressure (MAP) was significantly lower among SCI compared to naïve animals (17 ± 3 mmHg, *p* < 0.001). (**C**) Systemic vascular resistance (SVR) was significantly lower among SCI compared to naïve animals (0.11 ± 0.08 mmHg·min^−1^ µL^−1^, *p* = 0.034). (**D**): Heart rate (HR) was significantly higher among SCI compared to naïve animals (49 ± 17 beats per minute, BPM; *p* = 0.008).

Hemodynamic indices are presented in Table 1 and Figure 3. SBP, DBP, PP and MAP were all significantly reduced among SCI compared to naïve rats (all *p* < 0.001). HR was significantly higher among SCI rats compared to naïve rats (*p* = 0.008). Systemic vascular resistance (SVR) was also reduced in SCI rats compared to naïve (*p* = 0.038).

### 3.2. Left Ventricular Systolic Function Is Impaired in T3 300 kdyn SCI Rats

**Figure 4:**
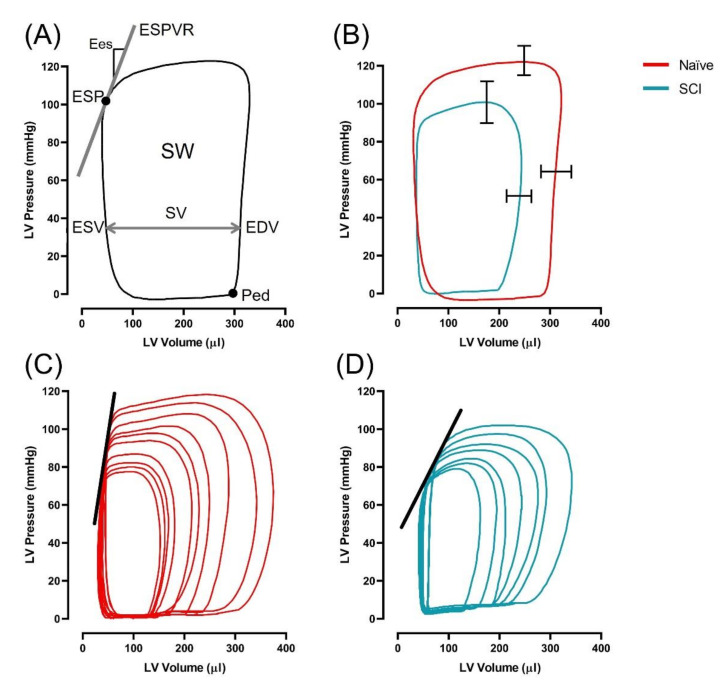
At 2 weeks post-SCI, animals underwent left ventricular catheterization to assess cardiac function. Pressure-volume analysis revealed reduced pressure, volume, and contractile function among SCI compared to naïve rats. (**A**) An example pressure-volume loop labelled with relevant indices acquired from pressure-volume analysis (ESV; end-systolic volume, EDV; end-diastolic volume, Pes; end-systolic pressure, Ped; end-diastolic pressure, SV; stroke volume, SW; stroke work (area of the pressure volume loop), ESPVR; end systolic pressure volume relationship, Ees slope of ESPVR). (**B**) Example basal pressure volume loop from SCI and naïve rats, overlaid with SEM bars, demonstrating diminished LV maximum pressure (22 ± 4 mmHg; *p* < 0.001) and EDV (50 ± 15 ul; *p* = 0.002) in SCI compared to naïve animals. C-D: Example inferior vena cava occlusions from naïve (**C**) and SCI (**D**) groups demonstrating reduced Ees among SCI compared to naïve animals (0.7 ± 0.1 mmHg/ul; *p* < 0.001).

LV measures of systolic and diastolic function are reported in Table 1 and select indices are displayed in Figure 4. Among SCI rats, a decrease in EDV (*p* = 0.002) and SV (*p* = 0.001) was observed compared to naïve rats, in the absence of changes to ESV (*p* = 0.256) and EF (*p* = 0.272). SW and SWI were significantly lower among SCI rats compared to naïve (both *p* < 0.001). Conversely, there was no difference in CO or CI between groups (*p* = 0.201; *p* = -0.610, respectively). Pes, Pmax and the maximum rate of rise of LV pressure (dP/dt_max)_ were lower among SCI compared to naïve rats (all *p* < 0.001). Measures of load-independent function, Ees (*p* < 0.001), dP/dt_max_−EDV (*p* < 0.001), and PRSW (*p* = 0.001) were significantly lower in SCI compared to naïve rats. Ea was not significantly different between groups (*p* = 0.296), however Ea/Ees was significantly higher in SCI rats compared to naïve.

For diastolic function, dP/dt_min_ was significantly lower among SCI rats compared to naïve (*p* > 0.001) in the absence of differences in end diastolic pressure (*p* = 0.231) and time constant of LV pressure decay (tau, *p* = 0.416).

### 3.3. Moderately-Severe T3 Midline Injury Interrupts Descending Pathways

**Figure 5:**
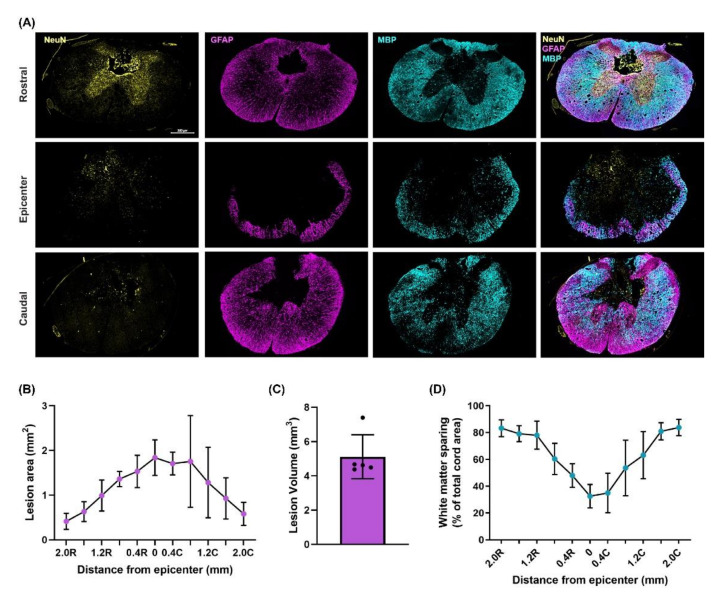
Lesion site characterization. (**A**) Representative immunohistological images of the rostral (top), epicenter (middle), and caudal (bottom) sections. Stains from left to right are Neuronal Nuclei (NeuN), Glial Fibrillary Protein (GFAP), Myelin Basic Protein (MBP), and the merged stain. Data were quantified every 400 µm from the lesion epicenter to a distance of 2 mm rostrally and caudally (n = 5). (**B**) The GFAP signal was used to quantify lesion area which reached an area of 1.75 ± 0.40 mm^2^ at the epicenter. Data points and bars represent the mean and standard deviations, respectively. (**C**) Lesion volume was calculated as Volume = Σ (area · section thickness · number of sections between samples) [24] and was found to be 4.26 ± 1.28 mm^3^. Bars represent the means and standard deviations overlaid with individual data. (**D**) White matter sparing was quantified using the MBP signal and reached a minimum sparing at the epicenter of 33 ± 9%. Data points and bars represent the mean and standard deviations, respectively.

**Figure 6:**
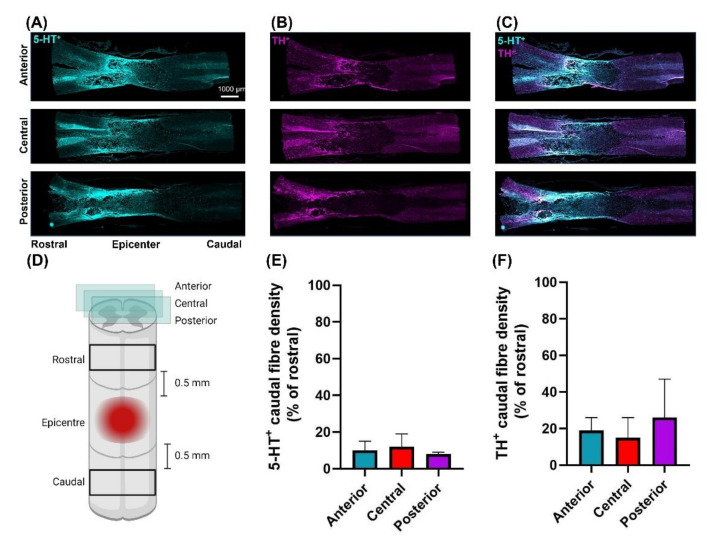
Representative immunohistological images of longitudinal spinal cord sections, anterior (top), central (middle), and posterior (bottom) (**A**–**C**). Stains from left to right are 5-HT^+^, TH^+^ and merged. Each quantified section was 500 µm removed from the one previous (**D**) Schematic depicting the anatomical location of where densities of 5-HT^+^ and TH^+^ were measured 0.5 mm rostral and caudal to the epicenter with the area of study being 2 mm wide by 1 mm tall. The associated density plots comparing the caudal to the rostral stain density across anterior, central and posterior sections for 5-HT^+^ (**E**) TH^+^ (**F**). Caudal sparing was quantified as 9 ± 2% and 18 ± 9% relative to rostral for 5-HT^+^ and TH^+^, respectively (n = 3). Data are presented as means ± standard error.

Following moderately-severe T3 SCI the lesion area was 1.75 ± 0.40 mm^2^ leaving 21 ± 6% tissue sparing. Lesion volume was 4.26 ± 1.28 mm^3^. White matter sparing at the epicenter was 33 ± 9% (Figure 5). The density of 5-HT^+^ fibres caudal to the epicenter was reduced to 9 ± 2% of rostral density (Figure 6). The density of TH^+^ fibres caudal to the epicenter were reduced to 18 ± 9% of the rostral density (Figure 6).

## 4. Discussion

We have developed a novel moderately severe high-thoracic midline contusion SCI model that produces robust and clinically relevant impairment in cardiac and hemodynamic function whilst preserving 33 ± 9% of white matter at the injury epicenter. Though our model also robustly reduces the density of 5-HT^+^ and TH^+^ fibres at and below the injury epicenter, we were able to clearly visualize both TH^+^ and 5-HT^+^ fibres projecting through and below the injury site. As such we believe this model provides a nice balance between producing a clinically relevant decline in CV function yet sparing sufficient bulbo-spinal sympathetic and serotonergic pathways that can be targeted with therapies designed to induce/alter spinal neuroplasticity with a view to improving CV function.

### 4.1. Resting Hemodynamics Are Impaired in T3 300 kdyn SCI Rats

Reduced SBP and MAP have been demonstrated in a variety of high-thoracic contusion, clip compression and transection SCI models. Though it is difficult to compare BP across studies due to heterogeneity in measurement technique and rodent strain, the magnitude of decline in SBP and MAP is typically in the 15–25 mmHg range with complete transection or severe contusion at the T2–T4 spinal level [24,26,27,32,34,37]. We found a similar 25 mmHg decline in the present study despite our model being less severe and exhibiting more sparing at the injury epicenter (see below) than those typically used to induce CV dysfunction. It has been recently shown that the major ˝hot-spot˝ for blood pressure control are the splanchnic projecting SPNs that exit the cord at the T11–T13 level [28]. SPNs in this region of the cord are under the control of both descending bulbo-spinal catecholaminergic and serotonergic [33] pathways originating in the RVLM and Raphe, respectively. Histological analyses of TH^+^ and 5-HT^+^ fibres in longitudinal sections of the spinal cord revealed our injury significantly reduces the density of both sets of fibers at and below (vs. above) the injury site. In turn, this loss of catecholaminergic and serotonergic excitatory input to SPNs reduces vascular tone, leading to splanchnic pooling and hypotension [38]. Persistent hypotension is of clinical importance as it contributes to the disproportionate burden of ischemic stroke heart disease observed in SCI [2]. Notably, a 20 mmHg decline in SBP and MAP is typical of that observed in individuals with chronic high-level SCI [39,40], thus increasing the potential for translation of findings using this model. Importantly, whilst TH^+^ and 5-HT^+^ fibre density was reduced post-SCI we were able to clearly visualize both TH^+^ and 5-HT^+^ fibres traversing the injury site. We believe the presence of such fibres, whilst insufficient to offset hypotension, can act as a target for neurotherapeutic interventions that aim to strengthen synaptic input.

### 4.2. Left Ventricular Systolic Function Is Impaired in T3 300 kdyn SCI Rats

Another major finding of the present was that almost all pressure- and volume-related indices of resting LV function were significantly decreased in SCI vs. naïve rats, with the exception of ESV and EF. We have previously reported similar findings in more severe models of SCI (i.e., T3 400 kdyn contusion with 5 s dwell, or T3 complete transection) [24,26,27,34,37], but not in less-severe models of SCI (T3 200 kdyn contusion, 5 s dwell) [34]. Reductions in volumetric function post-SCI also occurs clinically with high-level SCI. Although there was no difference in ESV, SV was lower in SCI rats likely due to reduced preload (i.e., EDV). Interestingly, this decrease in SV was compensated by increases in HR resulting in no statistical difference in CO between groups. A SCI-induced increase in HR has also been demonstrated in other studies with injuries at the T3–T5 spinal level, and has been hypothesized to result from increased sympathetic activity above the level of injury [41] as this injury model spares some sympathetic input to T1 level SPNs. It is equally possible, however, that SCI induces changes in cardiovagal balance such that HR can increase via vagal withdrawal sufficiently to normalize CO. Whilst the reduction in resting pressure and volume indices of LV systolic function are likely due to reduced catecholaminergic and serotonergic input to the SPNs in the T2–T5 level of the spinal cord [42], these indices are also critically dependent on changes in pre-load and afterload, both of which are impacted by SCI, as evidenced by reduced EDV and systemic blood pressure in this present study. Unlike resting pressure-volume indices, dP/dt_max_−EDV, PRSW and Ees obtained from IVC occlusions are largely insensitive to changes in load or rate [43] and as such are considered the reference metrics for LV systolic function [36]. We found all 3 metrics were significantly reduced in SCI vs. naïve rats, presumably due to the reduced density of 5-HT^+^ and TH^+^ fibres at and below the injury epicenter. The magnitude of reduction in these indices of contractility is similar to that observed in our more severe injury models [26,27,32,35] despite our current model sparing more white matter and there being a clear visualization of both TH^+^ and 5-HT^+^ fibres traversing the injury site. The reduction in Ees precipitated an increase in the Ea/Ees ratio, implying this SCI model impairs cardiac efficiency.

### 4.3. T3 Moderately-Severe Contusion Injury Interrupts Descending Pathways

Moderately-severe T3 spinal cord contusion injury resulted in substantial white matter damage at the epicenter and a lesion that extended at least 2 mm rostral and caudally encompassing the T2 spinal level thus interrupting supraspinal control to SPNs critical for CV regulation at the spinal levels described above. These histological findings are supported by the reduced density of 5-HT^+^ and TH^+^ fibres below the lesion suggesting that serotonergic and catecholaminergic input to SPNs is reduced by this model of SCI. Serotonergic [44,45] and catecholaminergic [46,47] bulbo-spinal fibres densely innervate areas of the cord associated with input to SPNs. Loss of supraspinal 5-HT^+^ fibres play a key role in inducing CV dysregulation including contributing to the development of AD and hypotension observed post-SCI [33,48]. Other models of SCI which have explored the relationship between 5-HT^+^ preservation and CV function post-SCI include complete crush [25], and partial transection [49] models of SCI at the T4 level. Neither crush injury nor partial transection reported reduced MAP despite showing reduced/absent density of serotonergic fibres caudal to the injury. While these SCI models were performed at a more caudal spinal level, CV dysfunction is seen in severe injuries as low as T6. This observation suggests, perhaps, that the mechanism of injury is not severe enough to sever sufficient pathways to impair CV function, given that it is necessary to decrease white matter sparing substantially to induce a decline in CV function post-SCI [24]. Other models of SCI which demonstrated motor recovery following ABT utilized a T10 contusive injury which resulted in 8–22% white matter sparing at the epicenter [17] or T10 hemisection models with approximately 35% white matter sparing [18]. Such levels of ’required’ sparing suggests that our model maintains sufficient pathways for successful application of therapies to induce neuroplasticity.

### 4.4. Comparison to Other Rodent Models of CV Instability

A number of rodent models of CV dysfunction have now been proposed in the literature and we have summarized the findings and gaps in knowledge from these models in Table 2. To aid in this comparison, we selected a number of key findings that we believe are critical for an animal model to exhibit when studying the efficacy of interventions aimed at inducing neuroplasticity. Our selected indices included reduced blood pressure, cardiac pressures and cardiac volumes, as well as sufficient tissue and white matter sparing at the epicentre and the presence of catecholaminergic and serotonergic fibres traversing the injury site. In the studies conducted to date that we are aware of, whilst almost all of the high-thoracic models induce reductions in blood pressure and/or heart function it is likely that only the current T3 contusion model has sufficient tissue sparing and catecholaminergic/serotonergic projections for such therapies to work. As such we believe our model achieves a feat not previously achieved in prior models; that is, modest tissue sparing at the injury epicenter yet a severe reduction in cardiac and hemodynamic function.

An additional benefit of this model is that animal health was greatly improved over our typical experience with more severe contusion and transection injury models. Notably, animals regained spontaneous voiding within 5 days post-injury compared to the typical 10 days seen among transected rats [52], returned to pre-injury health scores and pre-surgical body mass by 14 days-post injury and mortality was remarkably low with 93% of all animals surviving the initial injury surgery and no mortality across the 14-day period. We believe this model, therefore, improves upon animal welfare and decreases the burden of care on researchers.

### 4.5. Limitations

This model has yet to be tested in female rats to account for sex differences in autonomic function. Although we know that cardiac dysfunction manifests within the first 4 h post-SCI [31], and there are similar impairments at 3 and 7 days [53], 5 weeks [34], and 12 weeks post-SCI [32] the time course of CV function beyond 12 weeks in contusive models of SCI has not yet been characterized.

## 5. Conclusions

Here, we have presented a high-thoracic contusion model of SCI which demonstrated marked CV decline and modest tissue sparing at the epicenter, a feat not achieved by previous SCI models. Given the recent impetus of the field to move towards interventions that aim to enhance neuroplasticity (i.e., ABT and/or AIH) we believe this model will be useful to test the efficacy of these interventions to improve CV and autonomic function.

## Figures and Tables

**Table 1:** Anthropometric, hemodynamic and pressure-volume data for 2-week post T3 300 kdyn SCI and naïve rats.

	Naïve			SCI			*p*-Value
	Hemodynamic Data						
**SBP (mmHg)**	**121**	±	7	96	±	11	<0.001
DBP (mmHg)	70	±	7	58	±	9	<0.001
MAP (mmHg)	88	±	7	70	±	9	<0.001
PP (mmHg)	50	±	4	38	±	6	<0.001
HR (BPM)	413	±	38	462	±	48	0.008
SVR (mmHg·min^−1^µL^−1^) *	0.89	±	0.20	0.74	±	0.12	0.038
	**Pressure-Volume Data**						
ESV (µL)	66	±	18	59	±	11	0.256
EDV(µL)	311	±	47	261	±	23	0.002
**Systolic Function**							
SW (mmHg·mL)	33	±	7	21	±	4	<0.001
SWI (mmHg·mL^−1^100 g^−1^)	10.90	±	2.53	7.52	±	1.64	<0.001
CO (mL/min)	102	±	20	93	±	12	0.201
CI (mL·min^−1^100 g^−1^)	33.69	±	7.72	35.22	±	7.32	0.610
SV (µL)	245	±	33	202	±	24	0.001
Pes (mmHg)	98	±	11	75	±	10	<0.001
EF (%)	79	±	4	77	±	5	0.272
dP/dt_max_ (mmHg/s)	10316	±	809	6084	±	755	<0.001
Ees (mmHg/µL) **	1.59	±	0.23	0.89	±	0.24	<0.001
Ea (mmHg/µL)	0.41	±	0.09	0.38	±	0.07	0.296
Ea/Ees **	0.26	±	0.06	0.44	±	0.23	0.021
PRSW (mmHg) *	131	±	30	94	±	17	0.001
+dP/dt_max_–EDV (mmHg·s^−1^ µL^−1^) *	34	±	7	27	±	4	<0.001
**Diastolic Function**							
dP/dt_min_ (mmHg/s)	−5890	±	449	−4021	±	630	<0.001
Ped (mmHg)	3	±	2	4	±	4	0.231
τ (ms)	7.32	±	0.77	8.03	±	3	0.416

Data are presented as means ± SD. *p*-values represent significant difference following independent samples t test. SBP, systolic blood pressure; DBP, diastolic blood pressure; MAP, mean arterial pressure; PP, pulse pressure; HR, heart rate; SVR, systemic vascular resistance; ESV, end-systolic volume; EDV, end-diastolic volume; SW, stroke work; SWI, stroke work index; CO, cardiac output; CI, cardiac index; SV, stroke volume; Pes, end-systolic pressure; EF, ejection fraction; dP/dt_max_, maximum rate of rise of left ventricular pressure; Ees, end-systolic pressure-volume relationship; Ea, arterial elastance; PRSW, preload-recruitable stroke work; dP/dt_min_, maximum rate of decay of left ventricular pressure; Ped, end-diastolic pressure; τ time constant of left ventricular pressure decay. * denotes naïve n = 12, SCI n = 12, ** denotes naïve n = 12, SCI n = 9 due to difficulties in performing IVC occlusions in some animals.

**Table 2:** Comparisons with previous models of SCI that have been used to induce cardiovascular dysfunction.

	Injury Model					
	T3 300 kdyn Contusion	T2 400 kdyn Contusion	T2 200 kdyn Contusion	T2-3 Transection	T4 Complete Crush	T10 400 kdyn Contusion
↓ Blood pressure	✔	✔	✔	✔	✖	✖
↓ Cardiac pressures	✔	✔	?	✔	?	?
↓ Cardiac output	✖	✔	✖	✔	?	?
>15% tissue sparing	✔	✖	✖	✖	?	?
>20% white matter Preservation	✔	✖	✖	✖	?	✖
Preserved sub-lesional serotonergic/ catecholaminergic pathways	✔	✖	✔	?	✖	N/A
References		[24,26,27,34]	[24,34]	[23,30,32]	[25]	[50,51]

## Data Availability

Data available upon request.
